# Controlling the Temporal Structure of Brain Oscillations by Focused Attention Meditation

**DOI:** 10.1002/hbm.23971

**Published:** 2018-01-13

**Authors:** Mona Irrmischer, Simon J. Houtman, Huibert D. Mansvelder, Michael Tremmel, Ulrich Ott, Klaus Linkenkaer‐Hansen

**Affiliations:** ^1^ Department of Integrative Neurophysiology Center for Neurogenomics and Cognitive Research (CNCR), Amsterdam Neuroscience, VU Amsterdam Amsterdam 1081 HV Netherlands; ^2^ Bender Institute of Neuroimaging (BION), Justus Liebig University Giessen Giessen 35394 Germany

**Keywords:** absorption, criticality, long‐range temporal correlations, meditation

## Abstract

Our focus of attention naturally fluctuates between different sources of information even when we desire to focus on a single object. Focused attention (FA) meditation is associated with greater control over this process, yet the neuronal mechanisms underlying this ability are not entirely understood. Here, we hypothesize that the capacity of attention to transiently focus and swiftly change relates to the critical dynamics emerging when neuronal systems balance at a point of instability between order and disorder. In FA meditation, however, the ability to stay focused is trained, which may be associated with a more homogeneous brain state. To test this hypothesis, we applied analytical tools from criticality theory to EEG in meditation practitioners and meditation‐naïve participants from two independent labs. We show that in practitioners—but not in controls—FA meditation strongly suppressed long‐range temporal correlations (LRTC) of neuronal oscillations relative to eyes‐closed rest with remarkable consistency across frequency bands and scalp locations. The ability to reduce LRTC during meditation increased after one year of additional training and was associated with the subjective experience of fully engaging one's attentional resources, also known as *absorption*. Sustained practice also affected normal waking brain dynamics as reflected in increased LRTC during an eyes‐closed rest state, indicating that brain dynamics are altered beyond the meditative state. Taken together, our findings suggest that the framework of critical brain dynamics is promising for understanding neuronal mechanisms of meditative states and, specifically, we have identified a clear electrophysiological correlate of the FA meditation state.

## 1 INTRODUCTION

Meditation is frequently described as a form of mental training to cultivate cognitive capabilities, including attention, concentration, and emotion regulation (Lutz, Slagter, Dunne, & Davidson, 2008). With its popularity for attaining relaxation, for physical and mental health (review: Chiesa & Serretti, 2011), and enhanced awareness and absorption (Ott, 2007), there is increasing scientific interest in understanding the brain mechanisms involved (review: Tang, Hölzel, & Posner, 2015).

The history of meditation practice is rich and diverse, and, therefore, it is important to refrain from using “meditation” as an umbrella term, but instead specify which practice is used for greater experimental control (Tang et al., 2015). In this study, we work within the framework of the neurophenomenological matrix model (Lutz, Jha, Dunne, & Saron, 2015) according to which meditation practices differ on phenomenological dimensions (e.g., clarity, stability, object orientation). For example, open monitoring is a meditation where monitoring skills are transformed to a state of reflexive awareness with a broad scope of attention without focusing on one specific object (Lutz et al., 2015, 2008). In contrast, focused attention (FA) meditation is a concentrative practice with a well‐defined object such as the breath (Lutz et al., 2008). It entails the continuous focus on the given object, without distraction from internal (e.g., thoughts) or external (e.g., sounds) sources, resulting in a narrow aperture of focus with high clarity and stability (Lutz et al., 2015, 2008). Whenever the mind wanders or attention is drawn to another object, the meditator is supposed to redirect attention to the original target object. FA meditation is especially interesting because the cultivation of monitoring skills are necessary for many of the meditation types (Lutz et al., 2008) and particularly important for better control of mind‐wandering dynamics (Hasenkamp, Wilson‐Mendenhall, Duncan, & Barsalou, 2011). Concentrative, object‐based meditation techniques such as FA meditation can further be conceptualized as mental training to cultivate the faculty of absorption (Ott, 2003). Absorption reflects a person's ability to fully engage his or her attention in an experience (Wenk‐Sormaz, 2005), and can be seen as an accumulation of focused attention or attentional control (Grant et al., 2013). It is associated with openness to new emotional and cognitive experiences (Tellegen & Atkinson, 1974), leading to an increased ability to concentrate especially on inward experience (Pekala, Wenger, & Levine, 1985).

Functional magnetic resonance imaging (fMRI) during FA meditation has revealed correlated brain activations in areas related to voluntary regulation of attention, such as the prefrontal cortex, premotor cortex, and dorsal anterior cingulate cortex (Fox et al., 2016). In contrast, brain areas related to mind‐wandering, most notably the posterior cingulate cortex and the posterior parietal lobule of the default mode network, show relative deactivation (Buckner, Andrews‐Hanna, & Schacter, 2008; Hasenkamp et al., 2011). EEG target frequencies for attention modulation are in the alpha and beta range, since modulation of oscillations at those frequencies is connected to the facilitation of selective sensory gating (Jensen & Mazaheri, 2010), attentional suppression of distracting stimuli (Foxe & Snyder, 2011), detection of subtle tactile stimuli (Linkenkaer‐Hansen, Nikulin, Palva, Ilmoniemi, & Palva, 2004b), and signal‐to‐noise ratio of incoming sensory information (van Ede, de Lange, Jensen, & Maris, 2011). Indeed, FA meditation on mindful breathing has been associated with decreased spectral power in the beta frequency band (Saggar et al., 2012) and increased frontal alpha and occipital beta power in focused Zen meditation (Huang & Lo, 2009), but also with increased alpha and decreased theta spectral power during focus on breathing in the lower abdomen (Yu et al., 2011). This makes the uniform identification of spectral associations for FA meditation difficult. Therefore, in this study, we go beyond traditional EEG spectral analysis and test whether the temporal structure of brain oscillations, as reflected in the strength of long‐range temporal correlations (LRTC), is altered during FA meditation and after meditation training.

LRTC in neuronal oscillations are a well‐established property of oscillations emerging when networks are near the critical state (Hardstone et al., 2012; Linkenkaer‐Hansen, Nikouline, Palva, & Ilmoniemi, 2001). Critical‐state dynamics in the brain have been related to optimal information processing (Shew & Plenz, 2013) and the degree to which the brain remains capable of quick reorganization (Deco, Jirsa, & Mcintosh, 2013; Singer, 2013; Tognoli & Kelso, 2014) and, thus, is responsive to natural environments (Beggs & Plenz, 2003). It has been found that the temporal structure of these oscillations are influenced by performing a task (Irrmischer, Sangiuliano Intra, Mansvelder, Poil, & Linkenkaer‐Hansen, 2017; Palva & Palva, 2011, Palva, et al., 2013) and that these oscillations are associated with trial‐by‐trial variability in performance (He & Zempel, 2013; Linkenkaer‐Hansen, Nikulin, Palva, Kaila, & Ilmoniemi, 2004a), and dynamics of finger‐tapping errors (Smit, Linkenkaer‐Hansen, & de Geus, 2013). So far it has not been shown if these dynamics in neuronal oscillations are also influenced in the absence of an overt task, by the mere intention to switch our focus from a thinking resting state to a meditative focused attention state.

We hypothesized that attention is balanced at a point of instability between order and disorder, characteristic of so‐called critical systems, which allows transient focus and swift change. As practitioners restrain from distraction during meditation to maintain a single focus, we predict that they might experience a shift from more complex brain dynamics to a state of reduced information propagation and reduced temporal complexity of oscillations. Using data from two independent laboratories, we here show that the temporal complexity of neuronal oscillations is affected by FA meditation training. Furthermore, it is sensitive to the meditative state and the subjective experience of absorption as measured with the Tellegen Absorption Scale (Tellegen & Atkinson, 1974), a measure of full attentional engagement (Grant et al., 2013).

## 2 MATERIALS AND METHODS

### 2.1 Study 1

#### 2.1.1 Participants

Data were obtained at the VU Amsterdam from 8 healthy, experienced meditation practitioners (4 female, mean age: 41.7 years, *SD* = 6.7), with a minimum meditation practice of 5 years (*M* = 18 (*SD* = 10.7)), who were recruited through advertisement at local meditation schools that practice FA meditation on the breath (Zen and Vipassana). Additionally, data were obtained from 11 healthy but meditation‐naïve controls (9 female, mean age = 21.6 years, *SD* = 2.1).

#### 2.1.2 Measurements and design

EEG recordings were acquired using the *Electrical Geodesics EEG system* (GES200) with 128‐EGI HydroCel channel sponge‐base EEG‐caps at a sampling rate of 1000 Hz. Participants were measured during 5 min eyes‐closed rest (ECR) while sitting on a chair with the instruction “Please keep your eyes closed, relax, and try not to fall asleep,” followed by the Amsterdam Resting‐State Questionnaire (ARSQ) (Diaz et al., 2014, 2013), allowing the investigation of the content and quality of thoughts and feelings experienced during the experimental conditions. Statements were rated on a 5‐point Likert scale and, subsequently, the mean score of items belonging to each of the ten dimensions of thoughts and feelings were computed. Following the ECR experiment, participants performed five minutes of FA meditation (MED) with the instruction: “Focus on the sensations of breathing, without trying to change it. If you notice that the mind was wandering, try to return to the breath.” After the MED condition, the ARSQ was filled out again. In view of the high test–retest reliability of LRTC during ECR (Nikulin & Brismar, 2004), we decided it is feasible to keep the order of conditions constant to prevent possible carry‐over effects of meditation.

### 2.2 Study 2

#### 2.2.1 Participants

Data were obtained at the Bender Institute of Neuroimaging in Giessen from 20 healthy practitioners (10 female, mean age = 46.95 years, *SD* = 12.46) from different traditions, who had an average sitting practice of 3.7 days per week (*SD* = 1.7) for 36 minutes (*SD* = 12), but a great range of meditation experience ranging from 1 month to 33 years (M = 10.8 (*SD* = 10.2)), and 10 meditation‐naïve healthy controls (5 female, mean age = 41.4 years, *SD* = 14.44).

#### 2.2.2 Measurements and design

EEG was recorded inside an fMRI scanner using the *BrainVision Recorder* and the 32‐channel EASYCAP system (10–20 System, Jasper, 1958), including 2 EOG channels and ECG, with a sampling rate of 5000 Hz. After gradient artifact removal data had been down‐sampled to 250 Hz. Participants were measured during 20 min eyes‐closed rest (ECR), with the instruction to “lie passively and let thoughts flow without trying to concentrate on a specific topic.” This experiment was followed by 20 min of FA meditation (MED) with the instruction to “focus on the sensations of breathing around the nostrils without influencing the breath pattern, and returning your focus as soon as the mind was wandering off.” Meditation practitioners underwent the first year of the *Academy of Inner Science* (AIS) *Timeless Wisdom Training (TWT)*, which included a focus on concentrative meditation practices during the first year. The participants committed to follow the meditation practice on a daily basis. After this year, the measurements were repeated with both the practitioners and the controls. Immediately following the MED condition, participants filled in the Tellegen Absorption Scale (TAS) (Tellegen & Atkinson, 1974), a set of self‐report items that assesses an individual's trait absorption level in everyday life (five‐point Likert scale). One example for absorption is being so focused on the storyline while reading a book that the surroundings are completely forgotten.

#### 2.2.3 Cleaning procedure EEG

EEG data of both studies were preprocessed using EEGLAB (Delorme & Makeig, 2004) and the Neurophysiological Biomarker Toolbox (NBT; http://www.nbtwiki.net; Hardstone et al., 2012). Signals were bandpass‐filtered between 0.5 and 45 Hz using the Butterworth FIR‐filter implemented in Matlab. All signals were visually inspected in windows of 10 s and transient artifacts, for example, caused by head movements or eye blinks, and noisy channels were manually marked and omitted from the subsequent computations of spectral power and detrended fluctuation analysis (DFA). Typically, only 1–2 s around an artifact were marked. Subsequently, we re‐referenced the signals to the common average and applied independent component analysis (Infomax) (Bell & Sejnowski, 1995; Makeig, Jung, Ghahremani, & Sejnowski, 2000) for identification of independent components related to heartbeat or eye movements, which were visually identified and removed (on average only two components were removed). The remaining components were projected back to signal space. On average, 0.78% of time samples of the raw signals was removed due to sharp transient artifacts (Mdn = 0.78%, Min = 0%, Max = 5.63%) and the number of time samples removed was not different between conditions meditation (Mdn = 0.72%) and mind‐wandering (Mdn = 0.82%).

#### 2.2.4 Analysis

Before the analysis of power spectral density and long‐range temporal correlations, we down‐sampled the EEG signals from both studies to 250 Hz. This operation does not affect the power nor the temporal structure of oscillations in the five classical frequency bands investigated here (delta 1–4 Hz, theta 4–8 Hz, alpha 8–13 Hz, beta 13–30 Hz, and gamma 30–45 Hz). The power of oscillations was computed using the Welch method with a 4096‐point Hamming window and a frequency resolution of 0.25 Hz. The central frequency, $f_{c}$, was computed according to these formulas: $f_{c}=\frac{\sum_{f=f_{L}}^{f_{H}}fP(f)}{\sum_{f=f_{L}}^{f_{H}}P(f)},$ here $f_{L}$ and $f_{H}$ represent the lowest and highest frequency that defines a given frequency band, and P(f) denotes the power at frequency *f*.

**Figure 1:**
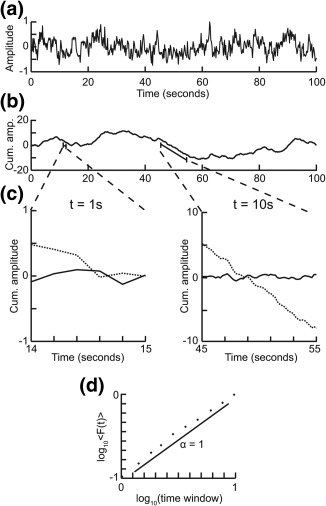
Step‐wise explanation of detrended fluctuation analysis. Adapted with permission from “Detrended fluctuation analysis: a scale‐free view on neuronal oscillations,” by R. Hardstone, S.S. Poil, G. Schiavone, R. Jansen, V. Nikulin, H.D. Mansvelder, and K. Linkenkaer‐Hansen, 2012*, Frontiers in physiology, 3*, 7. (a) Original time series. Taken from a 1/*f* signal sampled at 5 Hz with a duration of 100 s. (b) Cumulative sum of original signal shows large fluctuations away from the mean. (c) For each window size looked at, remove the linear trend from the signal, and then calculate the fluctuation. Signal shown as solid line and detrended signal shown as dotted line. (d) Plot the mean fluctuation per window size against window size on logarithmic axes. The DFA exponent is the slope of the best‐fit line (α = 1)

**Figure 2:**
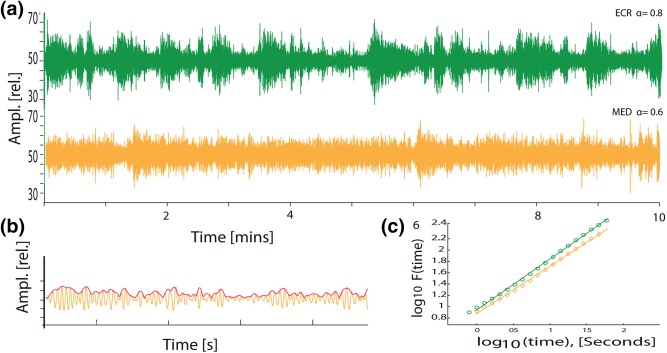
Beta oscillations exhibit greater complexity of amplitude modulations during eyes‐closed rest than during meditation. (a) Fractals are common in nature. These are complex physical structures exhibiting self‐similarity on multiple spatial scales. Adapted from Goldberger et al. (2002). Similarly, the temporal structure of a signal can be self‐similar in time. (b) Example of a 10‐min EEG from Cz filtered in the beta‐frequency range (13–30 Hz) during eyes‐closed rest (*green trace*) and during meditation (*yellow trace*) (data taken from Study 2). Note the change to more homogeneous variation in amplitudes during meditation compared to eyes‐closed rest, without change in average power. (c) Amplitude of beta oscillations (13–30 Hz) is shown in yellow and the amplitude envelope in red. (d) The grand average function of the DFA from all participants is plotted against window size in double‐logarithmic coordinates for channel Cz (α_ECR_ = 0.8 (±.03), α_MED_ = 0.73 (±.03)) [Color figure can be viewed at http://wileyonlinelibrary.com]

To quantify the strength of long‐range temporal correlations in the amplitude modulation of the oscillations, we used the detrended fluctuation analysis (DFA) (Peng, Havlin, Stanley, & Goldberger, 1995). In DFA, a signal profile is created by computing the cumulative sum of the time series (Figure 1a,b). The signal profile is then divided into time‐window sizes that are equidistant on a logarithmic scale, a local least‐square straight line is fitted to each of the windows, and the linear trend is removed (Figure 1c). Next, we calculate a fluctuation function which is defined as the average root‐mean‐square fluctuations of the integrated and linearly detrended signals for each time‐window size (with an overlap of 50% between windows). The fluctuation function is computed for all window sizes, plotted in double‐logarithmic coordinates, and a least‐square line fitted to the data (Figure 1d). The slope of the line represents the DFA exponent and an exponent of α = 0.5 indicates that the time series is uncorrelated, whereas 0.5 < α < 1 demonstrates a positive correlation in the signal. We applied this method to the amplitude envelope of each oscillation frequency band the way it was introduced by Linkenkaer‐Hansen et al. (2001) and it has been explained in detail with reference to open‐access Matlab scripts elsewhere (Hardstone et al., 2012). Thus, the EEG signal is first bandpass‐filtered and the Hilbert transform is then used to extract the amplitude envelope (Figure 2b,c). Next, the root‐mean‐square fluctuations of the integrated and linearly detrended signals, *F*(*t*), were calculated as a function of time window size, *t* (with an overlap of 50% between windows). As can be seen from the grand average DFA plot (Figure 2d), the DFA fluctuation function increased linearly in double‐logarithmic coordinates both during ECR and MED in agreement with previous studies using this analysis approach (for a review, see Hardstone et al., 2012). The DFA exponent is the slope of the fluctuation function *F*(*t*) in a given interval, which was set to 5–50 s for delta and theta band, from 2 to 50 s for alpha and 1 to 50 s for the beta and gamma bands. The lower time scale of fitting the power‐law was set higher for slow oscillations to exclude temporal autocorrelations introduced by the bandpass filters. The upper end of the fitting interval was set to 50 s because it is generally recommended to incorporate 6–10 independent windows of the longest time scale investigated to avoid noisy estimates of the DFA exponent (Hardstone et al., 2012; Hu, Ivanov, Chen, Carpena, & Stanley, 2001). Note that we did not compare exponents of signals of different length, and applications of DFA have also been used with fits longer than half the signal length (Palva et al., 2013). In practice, the only minimum duration of a signal for which one can compute the DFA exponent is the size of the largest time window (i.e., 50 s in our case).

Parametric tests (paired‐samples *t* tests, significance level: *p* < .05) were used in both studies for possible effects of condition (ECR vs MED) within participants and between. Further, correlations between questionnaire scores and DFA were calculated. For an analysis of covariance with demographic variables (age and gender) an ANCOVA model was computed in R (version 3.3.1). Tests were performed per channel, and binomial multiple‐comparisons corrected (Montez et al., 2009; Poil et al., 2011). The binomial multiple comparisons correction tests whether a significant number of channels reaches the significance level of *p* < .05 within a specific frequency band. The likelihood of having 12 channels out of 128 (Study 1) or 5 channels out of 30 (Study 2) by chance is given by the binomial distribution to be <5%, cf. (Montez et al., 2009; Nikulin, Jönsson, & Brismar, 2012; Schiavone et al., 2014). All DFA exponents reported in the main text are averages of significant electrodes across subjects.

## 3 RESULTS

### 3.1 Results of Study 1

We compared cognitive state and neuronal oscillations during 5 min of eyes‐closed rest (ECR) and 5 min of FA meditation (MED) in a group of meditation practitioners and a group of meditation‐naïve participants (Methods). Measurements were done in the lab in Amsterdam, The Netherlands.

#### 3.1.1 Thoughts and feelings during rest and meditation

**Figure 3:**
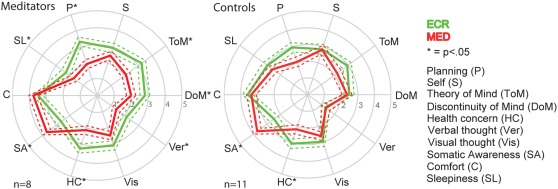
Meditation affects the content and quality of thoughts and feelings. Spider plots of thought dimensions measured using the ARSQ show differences between ECR (*green*) and MED (*red*). Meditation reduced thoughts about theory of mind, planning, sleepiness, verbal thought, thinking about health, and discontinuity of mind, while increasing somatic awareness in practitioners. The control group showed reduced health concern and increased somatic awareness. Stars represent significance of the difference in ARSQ dimension between ECR and MED: *p < .05; the dashed line represents the standard error of the mean [Color figure can be viewed at http://wileyonlinelibrary.com]

To investigate whether cognitive changes emerge during meditation compared to the classical eyes‐closed resting‐state condition, where no restrictions for the focus of thoughts are given, the participants of Study 1 filled in the Amsterdam Resting‐State Questionnaire (ARSQ), both immediately after the ECR and after the MED condition. During MED, the practitioners experienced less Theory of Mind (*M*_ECR_ = 2.2, *M*_MED_ = 1.1, *t*(7) = −4.1, *p* = .007), Planning (*M*_ECR_ = 2.3, *M*_MED_ = 1, *t*(7) = −3.7, *p* = .01), Sleepiness (*M*_ECR_ = 1.3, *M*_MED_ = 0.7, *t*(7) = −2.8, *p* = .03), Verbal Thought (*M*_ECR_ = 1.1, *M*_MED_ = .04, *t*(7) = −2.9, *p* = .026), Health Concern (*M*_ECR_ = 2.3, *M*_MED_ = 1.1, *t*(7) = −3.6, *p* = .011) and Discontinuity of Mind (*M*_ECR_ = 1.81, *M*_MED_ = 1, *t*(7) = −2.3, *p* = .059) compared to ECR, while Somatic Awareness (*M*_ECR_ = 1.6, *M*_MED_ = 2.7, *t*(7) = 3.9, *p* = .026) increased, in line with the objective of taking the breath as the focus of attention (Figure 3). The control group decreased in Health Concern (*M*_ECR_ = 2.1, *M*_MED_ = 1.2, *t*(10) = −2.7, *p* = .022), and also increased in Somatic Awareness (*M*_ECR_ = 1.5, *M*_MED_ = 2.7, *t*(10) = 3.8, *p* = .003 (Figure 3).

#### 3.1.2 Meditation reduces the complexity of neuronal oscillations during meditation

**Figure 4:**
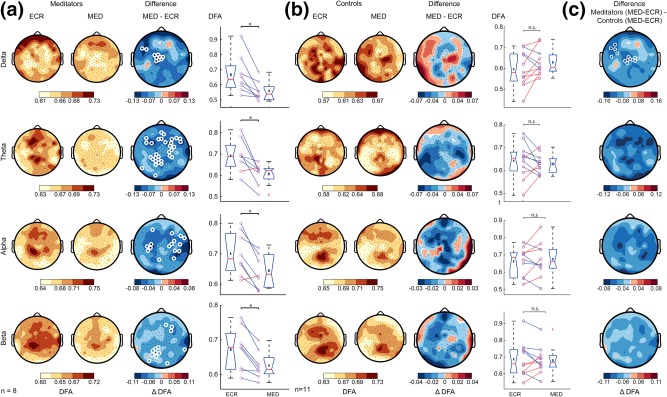
Study 1: Only meditation practitioners exhibit weaker LRTC during meditation compared to rest. (a) Grand‐average topographies of DFA are shown for ECR (first column) and MED (second column), and the difference of MED minus ECR (third column). Fourth column shows the average DFA exponent across significant electrodes for each subject. The delta‐band did not pass the multiple‐comparison criterion, but the topography speaks for the same trend as theta, alpha, and beta. (b) The same plots as in (a) are shown for the control group. (c) Grand‐average topographies comparing the effect of condition in the meditator and control groups. The difference was calculated as meditators (MED‐ECR) minus controls (MED‐ECR). The rows display the DFA for the delta (1–4 Hz), theta (4–8 Hz), alpha (8–13 Hz), and beta band (13–30 Hz), respectively. White circles denote channels with *p* < .05 (*t*‐test, binomial multiple comparison corrected). Meditation reduces the complex dynamics of neuronal oscillations in experienced practitioners, but not in controls [Color figure can be viewed at http://wileyonlinelibrary.com]

To test the effect of meditation on the temporal structure and stability of neuronal oscillations, we computed the DFA exponent as an index of LRTC. Paired‐samples *t* tests were conducted to compare the difference in DFA between ECR and MED for meditation practitioners and meditation‐naïve controls within each frequency band (Figure 4). Compared to ECR, practitioners showed a decrease of DFA during meditation for theta (α_ECR_ = .69 ± .03, α_MED_ = .61 ± .02, *t*(7) = −3.4, *p* = .01), alpha (α_ECR_ = .70 ± .02, α_MED_ = .64 ± .02, *t*(7) = −3.7, *p* = .008), and beta (α_ECR_ = .67 ± .02, α_MED_ = .63 ± .01, *t*(7) = −4.2, *p* = .004) frequency bands and a trend for the delta band (Figure 4a). These changes in DFA between ECR and MED were widespread across the scalp but were most pronounced above parietal, central and frontal regions (Figure 4a). Importantly, these effects were not present in meditation‐naïve controls, suggesting that meditation training influenced the ability to control temporal structure of neuronal oscillations during this FA meditation task (Figure 4b). The DFA values in the boxplots of Figure 4a,b show average DFA exponent values across all significant channels. In addition to within‐group analyses, a group comparison was carried out to investigate differences in temporal structure of oscillations between meditation practitioners and controls. Independent‐samples *t* tests were conducted to compare the difference in DFA between ECR and MED for meditators with the corresponding difference in DFA for controls within each frequency band. DFA decreased more for meditators compared to controls, although the effect was significant after binomial multiple comparisons correction only for DFA within the delta band (Δα_Meditators_ = −0.10 ± 0.10, Δα_Controls_ = 0.01 ± 0.05, *t*(17) = 3.2, *p* = .006) (Figure 4c). A one‐way analysis of covariance (ANCOVA) was then used to determine whether this difference in change in delta‐band DFA was significant when controlling for age and gender. After controlling for age and gender, there was no significant effect of group on change in DFA for the delta frequency band (Supporting Information, Figure 1).

### 3.2 Results of Study 2

#### 3.2.1 Independent sample confirms that meditation was associated with reduced LRTC

**Figure 5:**
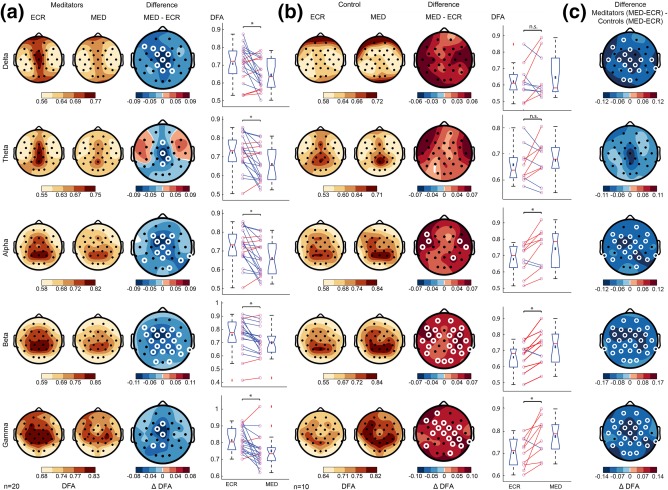
Study 2: Changes in DFA for practitioners and meditation‐naïve controls from ECR to MED. (a) Grand‐average topographies of DFA values are shown for ECR (first column) and MED (second column), and the difference of MED minus ECR (third column). Fourth column shows the average of significant electrodes for each subject. (b) The same plots as in (a) shown for the controls participants. (c) Grand‐average topographies comparing the effect of condition in the meditator and control groups. The difference was calculated as meditators (MED‐ECR) minus controls (MED‐ECR). The rows display the DFA for the delta (1–4 Hz), theta (4–8 Hz), alpha (8–13 Hz), beta (13–30 Hz), and gamma band (30–45 Hz), respectively. White circles denote channels with *p* < 0.05 (*t* test, binomial multiple comparisons corrected). In practitioners, complex dynamics of neuronal oscillations are reduced during meditation. The changes are significant in 5 frequency bands, showing a global state effect with central locations. The control group has increased LRTC during meditation [Color figure can be viewed at http://wileyonlinelibrary.com]

In parallel to Study 1 in Amsterdam, an independent EEG study on practitioners and meditation‐naïve participants was done in Giessen, Germany. This study had increased power due to a larger sample size and longer recording time for the EEG. Similar to Study 1, participants were recorded during eyes‐closed rest (ECR) followed by 20 min of FA meditation (MED). Paired‐samples t‐tests were conducted to determine within‐group differences in DFA between ECR and MED. In agreement with Study 1, LRTC were decreased during MED compared to ECR for meditation practitioners for multiple frequency bands (Figure 5). DFA in the delta (α_ECR_ = .71 ± .02, α_MED_ = .64 ± .02, *t*(19) = −2.4 *p* = .025), theta (α_ECR_ = .72 ± .02, α_MED_ = .66 ± .02, *t*(19) = −2.7, *p* = .013), alpha (α_ECR_ = .73 ± .03, α_MED_ = .68 ± .03, *t*(19) = −2.7, *p* = .015), beta (α_ECR_ = .76 ± .03, α_MED_ = .7 ± .03, *t*(19) = −2.8, *p* = .011), and gamma (α_ECR_ = .81 ± .01, α_MED_ = .75 ± .02, *t*(19) = −2.7, *p* = .013) band decreased during meditation with a widespread and Cz‐centered scalp topography, suggesting a global change of brain state (Figure 5a). Interestingly, meditation‐naïve controls did not show a reduction but rather showed an *increase* of LRTC during MED for alpha (α_ECR_ = .68 ± .03, α_MED_ = .74 ± .04, *t*(9) = 2.6, *p* = .027), beta (α_ECR_ = .66 ± .03, α_MED_= .72 ± .03, *t*(9) = 2.9, *p* = .018), and gamma (α_ECR_ = .70 ± .02, α_MED_ = .76 ± .02, *t*(9) = 2.7, *p* = .023) frequency bands (Figure 5b). Again, independent‐samples *t* tests were used to investigate differences in LRTC between meditators and controls. The decrease in DFA from ECR to MED was larger for meditators compared to controls for delta (Δα_Meditators_ = −0.05 ± 0.09, Δα_Controls_ = 0.04 ± 0.10, *t*(28) = 2.7, *p* = .013), alpha (Δα_Meditators_ = −0.04 ± 0.08, Δα_Controls_ = 0.04 ± 0.07, *t*(28) = 3.1, *p* = .005), beta (Δα_Meditators_ = −0.05 ± 0.07, Δα_Controls_ = 0.06 ± 0.05, *t*(28) = 4.5, *p* = .0001), and gamma (Δα_Meditators_ = −0.04 ± 0.08, Δα_Controls_ = 0.07 ± 0.08, *t*(28) = 3.3, *p* = .002) frequency bands (Figure 5c). After controlling for age and gender using a one‐way ANCOVA, the effect of group remained significant for all frequency bands: delta (*F*(1,24) = 9.3, *p* = .006), alpha (*F*(1,24) = 8.6, *p* = .007), beta (*F*(1,24) = 18.5, *p* = .0002), and gamma (*F*(1,24) = 10.1, *p* = .004) (Supporting Information, Figure 2).

#### 3.2.2 Suppression of LRTC during FA meditation is related to duration of meditation and absorption

**Figure 6:**
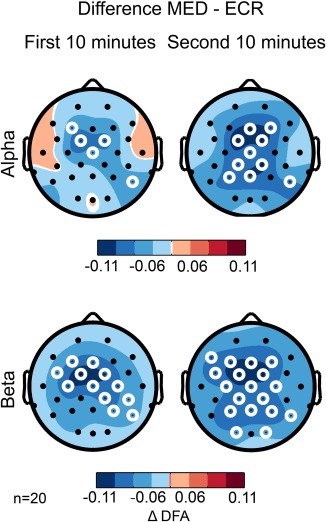
Meditation‐related reduction in LRTC is more pronounced in the second half of experiments. Splitting the recordings into first and second 10 minutes shows that the difference in DFA between MED and ECR is larger for practitioners in the second half (second column) compared to the first half of the recordings (left column) in the alpha (8–13 Hz) and beta (13–30 Hz) bands. White circles denote channels with *p* < 0.05 (*t* test, binomial multiple comparisons corrected) [Color figure can be viewed at http://wileyonlinelibrary.com]

Given the fairly long 20‐min period of meditation, we next asked if the suppression of LRTC could be used to monitor changes in the meditative state. First, we split the 20 min MED and ECR recordings of the practitioners into two 10‐min segments and analyzed those separately. The mean reduction of LRTC over all electrodes in the MED condition relative to ECR was stronger in the second half compared to the first half for alpha (Δα = −.0227 ± .001 to Δα = −.0522 ± .001 (*t*(29) = −11.46, *p* ≤ .0001)), and for beta oscillations (Δα = −.0432 ± .023 to Δα = −.0593 ± .019 (*t*(29) = −7.78, *p* ≤ .0001) (Figure 6).

**Figure 7:**
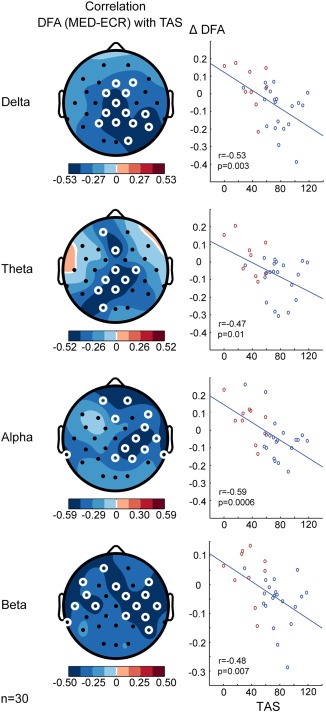
DFA is negatively correlated to the Tellegen Absorption Scale. Topographies show correlation between scores on the TAS and the difference in DFA exponents between MED and ECR for delta (1–3 Hz), theta (4–8 Hz), alpha (8–13 Hz), and beta (13–30 Hz) frequency bands. Both practitioners (blue) and controls (red) are included in this analysis. White circles denote channels with *p* < .05 (paired‐samples *t* test, binomial multiple comparisons corrected). Participants who report higher trait absorption show more suppression of LRTC during meditation [Color figure can be viewed at http://wileyonlinelibrary.com]

Next, we correlated the Tellegen Absorption Scale (TAS) with the change in LRTC from ECR to MED to test whether the suppression of temporal structure in neuronal oscillations was associated with the individual experiences of absorption. We observed negative associations in the delta (*r*(28) = −.53, *p* = .003), theta (*r*(28) = −.47, *p* = .01), alpha (*r*(28) = −.59, *p* = .0006), and beta bands (*r*(28) = −.48, *p* = .007) (Figure 7). This is adding subjective evidence to the notion that the observed reductions in LRTC are related to the ability to produce a stable state of focused attention.

#### 3.2.3 Training reinforces meditation‐induced suppression of complex brain dynamics

To test whether the ability to reduce LRTC of oscillations during meditation is a “skill” that the practitioners have to begin with, or if it could be developed further through training, the practitioners were measured again after participating in a one‐year meditation training with a focus on concentrative meditation practices, which the participants committed to in the form of daily meditation (see Methods). After the training, the reported meditation frequency increased from 3.7 (*SD* 1.7) to 4.7 (*SD* 1.6) days per week, (*t*(13) = 2.1, *p* = .028, one‐tailed). The meditations lasted on average for 37.3 (*SD* 11.6) min, ranging from a minimum of 15 to a maximum of 60 min.

**Figure 8:**
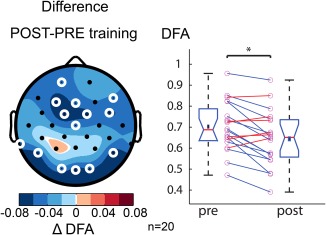
One year of meditation training is associated lower LRTC during meditation. The topography shows the difference in DFA exponents during meditation before and after one year of meditation training in the alpha band (8–13 Hz). White circles denote channels with *p* < .05 (*t* test, binomial multiple comparisons corrected). Right, the average DFA exponent across significant electrodes for each subject at the intake (pre) and after one year of training (post). After the training, the LRTC of alpha oscillations during MED is reduced. The Greek delta symbol is used to denote a “difference.” [Color figure can be viewed at http://wileyonlinelibrary.com]

Intriguingly, LRTC during MED decreased from the first to the second measurement in all frequency bands, albeit reaching significance only in alpha (α_MED1_ = .70 ±.03, α_MED2_ = .64 ±.03, *t*(19) = −2.5, *p* = .02), suggesting that participants became more skilled at entering a functional state characterized by suppressed LRTC (Figure 8).

#### 3.2.4 Meditation also affects the eyes‐closed rest brain dynamics

The strength of LRTC in eyes‐closed rest EEG is a trait‐like phenomenon, stable over time and strongly influenced by genes (Linkenkaer‐Hansen et al., 2007; Nikulin & Brismar, 2004). Our data confirmed this as in none of the analyzed frequency bands did we observe a change in LRTC reaching the *p* < .05 level of significance (*data not shown*) in the control group after one year delay.

**Figure 9:**
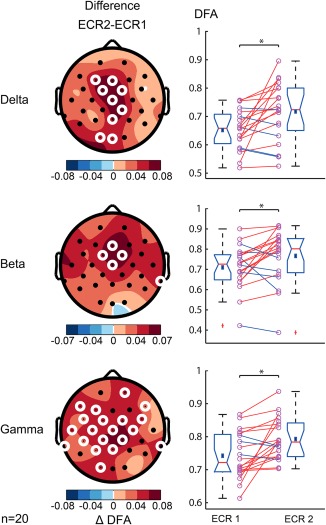
One year of meditation training is associated with increased resting‐state LRTC in multiple frequency bands. Scalp topographies show post‐ minus pretraining resting‐state LRTC for delta (1–3 Hz), beta (13–30 Hz), and gamma (30–45 Hz) frequency bands. White circles denote channels with *p* < .05 (paired‐samples *t* test, binomial multiple comparisons corrected). Right, the average of significant electrodes for each subject for ECR before (ECR1) and ECR after training (ECR2) are shown. LRTC during resting state increases after one year of meditation training [Color figure can be viewed at http://wileyonlinelibrary.com]

In contrast, the practitioners showed an increase of LRTC in the ECR recordings after the training in the delta (α_ECR1_ = .65 ±.02, α_ECR2_ = .72 ±.02, *t*(19) = 2.6, *p* = .016), beta (α_ECR1_ = .71 ± .02, α_ECR2_ = .77 ± .03, *t*(19) = 2.4, *p* = .029), and gamma bands (α_ECR1_ = .74 ± .02, α_ECR2_ = .79 ± .01, *t*(19) = 2.5, *p* = .022) (Figure 9). This suggests that in addition to the short‐term state effect of FA meditation (Figure 5), regular practice also has long‐term influences on the brain, even when not engaged in meditation.

#### 3.2.5 Additional EEG analysis: Meditation‐induced changes in spectral power and Central frequency

Finally, we investigated possible changes in spectral power and central frequency to compare our study with existing literature. In Study 1, none of these measures reached significance. In Study 2, we replicated the findings that FA meditation is associated with a widespread increase in alpha power in frontal, central, parietal and occipital locations (*M*_ECR_ = 113 ±17 μV^2^/Hz, *M*_MED_ = 136 ± 19 μV^2^/Hz, *p* = .03) (Yu et al., 2011), and a decrease in alpha central frequency oscillations in frontal and parietal regions (*M*_ECR_ = 9.92 ±.07 Hz, *M*_MED_ = 9.78 ±.08, *t*(19) = −2.8, *p* = .011), which is in line with the decrease in individual alpha frequency reported previously (Saggar et al., 2012). It is important to stress that DFA was sensitive in both studies, even with the smaller sample size and recording duration of Study 1. Taken together, our data suggest that LRTC of oscillations is a sensitive biomarker for monitoring short‐ and long‐term effects of FA meditation.

## 4 DISCUSSION

To the best of our knowledge, this study presents the first application of temporal correlation analysis of neuronal oscillations during FA meditation. We measured ongoing EEG and observed in two independent datasets that prior experience with FA meditation was associated with reduced long‐range temporal correlations (LRTC) during the meditation period compared to the eyes‐closed rest condition. Additionally, Study 2 showed that this reduction was stronger in the second half of the 20‐min meditation session, enhanced by an additional 1 year of meditation training, and correlated with the subjective experience of absorption. We also found that regular practice can increase resting‐state LRTC.

In Study 1, the transition from eyes‐closed rest (ECR) to FA meditation (MED) showed a reduction of LRTC for theta (4–8 Hz), alpha (8–13 Hz), and beta (13–30 Hz) frequency bands. Importantly, this change was not observed in the control group of meditation‐naïve subjects. The between‐group test showed that in the meditators the DFA reduced more than in the controls in all frequency bands, but this effect only passed multiple comparisons correction in the delta band, possibly due to too little power caused by the small sample size.

In light of recent discussions about replicability in research (Open Science Collaboration, 2015) it is important to acknowledge that these findings were replicated and more pronounced in Study 2, which included more participants and longer recordings. Here, the within‐subject reduction of LRTC reached significance in all five frequency bands measured (delta, theta, alpha, beta, gamma), indicating a change in global brain state. Although the number of channels in the two studies differed, and minor differences in scalp topographies were visible, one should note the similarities in terms of scalp‐wide reductions in LRTC in both studies. In Study 2, the between‐group test showed again that in the meditators the DFA reduced more than in the controls in all frequency bands, passing multiple comparisons correction in the delta, alpha, beta, and gamma frequency bands. An analysis of covariance showed that the demographic factors age and gender did not influence the reduction in LRTC. This is in line with observations that LRTC in these brain oscillations have been shown to remain stable throughout adulthood (Smit et al., 2011) and show no significant gender effects at the single‐electrode level (Nikulin & Brismar, 2005).

A surprising result is the increase in LRTC found for the alpha, beta, and gamma frequency bands in the controls of study 2 while the control group in study 1 did not show any significant change. A possible explanation could be the difference in the duration of experiments (5 min vs 20 min): The ARSQ showed that in experiment 1 the control participants increased in “Somatic Awareness” during the meditation condition, suggesting that it is possible for inexperienced participants to follow the instruction to focus on the body sensation of breathing. In Study 2, the inexperienced participants may have found it challenging to maintain—or even increase—attentional focus for 20 minutes. As a consequence, frequent switching between focusing on the sensations of breathing and mind‐wandering may have occurred. This, in turn, may have resulted in more complex variability in oscillatory brain activity and a higher DFA exponent in the control cohort, which is analogous to recent findings of reduced LRTC when subjects successfully attend to a sustained visual attention task (Irrmischer et al., 2017).

However, what could be the functional role of the reduction in LRTC associated with FA meditation? For optimal functioning, the brain needs to adapt in response to internal and external demands. For this, neuronal activity is required to organize on different topological and temporal scales in order to produce a certain brain state, which is capable of adapting to the requirements at hand (Fries, 2005; Singer, 2013; Varela, Lachaux, Rodriguez, & Martinerie, 2001). LRTC allow organization at multiple topological *and* temporal scales, which in “normal” waking consciousness are observable near the critical state (Linkenkaer‐Hansen et al., 2001). This is pointing toward an adapted response to a natural environment which displays many critical features (Beggs & Plenz, 2003). If the demands change, in this case, an alteration from normal waking conscious state to FA meditation (i.e., “one‐pointed focused attention”), information propagation ultimately needs to be reduced. This fits with the neurophenomenological matrix model (Lutz et al., 2015) in which FA meditation is associated with a narrow aperture of focus, clarity, and stability, whereas mind‐wandering—which is opposing FA meditation—is related to low stability. Increased perceptual stability has indeed been observed after and during FA meditation (Carter et al., 2005). Similarly, increased performance stability in attention tasks were found after an intensive meditation retreat (Lutz et al., 2009; Zanesco, King, Maclean, & Saron, 2013).

Last, it has been argued that less temporal redundancy leads to more efficiency in processing (He, 2011), perhaps enhancing the conscious experience of the object of focus. Indeed, the ARSQ showed that the practitioners decreased in nearly all dimensions of resting‐state cognition, but increased in somatic awareness, the focus of their attention.

### 4.1 Long‐term effects of regular practice

The longitudinal design, with a measurement before and after one year of meditation training, provided the unique opportunity to test whether mental training can directly influence these brain dynamics. Comparing LRTC during the meditative state before and after the training showed a trend towards further attenuating LRTC in all frequency bands, albeit significant only in the alpha band. This indicates that the ability to influence brain dynamics through FA meditation is a skill that may be learned and enhanced through regular practice. Another striking observation from our data was the change in resting‐state EEG in practitioners. The strength of LRTC in eyes‐closed rest EEG is a trait‐like phenomenon, which is both significantly heritable (Linkenkaer‐Hansen et al., 2007) and stable over time (Nikulin & Brismar, 2004). The control group did not follow any intervention or training and therefore as expected showed no change in resting‐state brain dynamics. The practitioners on the other hand showed an increase of LRTC in delta, beta, and gamma bands during normal rest after a one‐year meditation training. This observation shows a lasting impact of regular practice on normal waking state consciousness and brain dynamics, even without engaging in meditation. This observation is adding to the cumulative evidence of lasting effects of meditation on daily life (Chan & Woollacott, 2007; Frewen, Lundberg, MacKinley, & Wrath, 2011). From a dynamic perspective, it is very intriguing that regular practice showed the dichotomy of increased LRTC during resting state, while at the same time enhancing the ability to create a state of reduced LRTC during FA meditation. This shows an increase in the total dynamical *range* of neuronal oscillations in the practitioners.

### 4.2 LRTC and absorption

Attentional absorption as measured with the TAS can be described as the tendency to have “episodes of ‘total’ attention that fully engage one's representational resources” (Tellegen & Atkinson, 1974). Practitioners have a greater tendency to be absorbed in experiences, report higher absorption scores using this scale (Davidson, Goleman, & Schwartz, 1976; Hölzel & Ott, 2006), and people with high absorption level can reach deeper meditative states more quickly (Hölzel & Ott, 2006). Further, a higher absorption level is associated with thicker gray matter in nodes of the cingulo‐fronto‐parietal attention networks (Grant et al., 2013), suggesting a relationship between absorption and attentional control. Additionally, absorption scores of practitioners have been found to be related to the frequency of their practice (Hölzel & Ott, 2006), suggesting the trait may be sensitive to training. Our data show that the participants that showed a larger reduction in LRTC during meditation are also the ones who report higher trait absorption.

### 4.3 Possible mechanisms causing the change in LRTC during meditation

In light of a theory development explaining potential underlying neuronal correlates causing the reduction in LRTC during FA meditation, we are looking at mechanisms related to global changes in cortical dynamics both in terms of anatomical locations (scalp regions) and frequency bands. Therefore, we speculate that the target mechanisms are neuromodulators, and changes in the thalamus and the cingulate cortex activity.

Neuromodulators have been shown to influence global neuronal fluctuations by balancing the excitation/inhibition ratio within the signal. For example, an increase in serotonin is related to a shift toward excitability (Moreau, 2010) and has been observed during Zen practice (Yu et al., 2011). GABA, on the other hand, leads to inhibition (Shew, Yang, Petermann, Roy, & Plenz, 2009), and increases have been found after meditation (mindfulness/vipassana) (Guglietti, Daskalakis, Radhu, Fitzgerald, & Ritvo, 2013) and yoga practice (Streeter et al., 2007; Streeter et al., 2010). Based on computational models of LRTC (Poil, Hardstone, Mansvelder, & Linkenkaer‐Hansen, 2012), one would expect a meditation‐related increase in GABA to drive neuronal dynamics toward a subcritical regime characterized by lower DFA exponents as we observed it.

Another possible driver for widespread changes in cortical stability could be thalamic activity in the form of slow underlying oscillations, typically visible in central scalp locations (Steriade, 2006). Thalamic input has long been associated with selective attention (Crick, 1984) and alertness (Halassa et al., 2011; Steriade, McCormick, & Sejnowski, 1993) by integrating the competition between different inputs at the thalamic level itself (Lam & Sherman, 2011). Indeed, thalamic involvement in FA meditation has repeatedly been hypothesized (Austin, 2013; Guglietti et al., 2013; Kerr, Sacchet, Lazar, Moore, & Jones, 2013; Newberg & Iversen, 2003) and, recently, successfully modeled (Saggar et al., 2015).

Last, strongly connected to the thalamus is the highly active and anatomically widely connected cingulate cortex (Hagmann et al., 2008). Here, especially the posterior cingulate cortex is related to attention regulation in terms of broadening or narrowing of the focus and its direction toward internal versus external sources of information (Fox et al., 2005; Leech, Kamourieh, Beckmann, & Sharp, 2011). Possibly acting as a “transmodal thalamocortical hub,” it is exhibiting complex interactions across the whole brain, including attentional networks, and therefore tuning the meta‐stability of the system according to demands (Leech & Sharp, 2014). Indeed, increased functional connectivity has been observed during meditation (Brewer et al., 2011) and PCC deactivation has been used successfully in open monitoring meditation neurofeedback (Garrison et al., 2013; Lutterveld et al., 2016).

### 4.4 Outlook

According to the neurophenomenological matrix model, meditation practices differ according to phenomenological dimensions. For example, Open Monitoring meditation, which is similar to FA meditation in high clarity and stability, has larger aperture and less object orientation (Lutz et al., 2015). Thus, it could be interesting to compare the effect of other meditation types on brain complexity. Still, from the perspective of identifying a physiological indicator of meditation‐related changes in brain states, we see the DFA exponent to be very promising.

## CONFLICT OF INTEREST

The authors declare no competing financial interests.

## Supporting information

Additional Supporting Information may be found online in the supporting information tab for this article.

Supporting Information Figure 1

Supporting Information Figure 2

Supporting Information Figure 1
