# Supplementary figures and images for: Controlling the Temporal Structure of Brain Oscillations by Focused Attention Meditation
Source: Hum Brain Mapp. 2018 Jan 13;39(4):1825–38. doi: 10.1002/hbm.23971 (PMC6585826; doi:10.1002/hbm.23971)

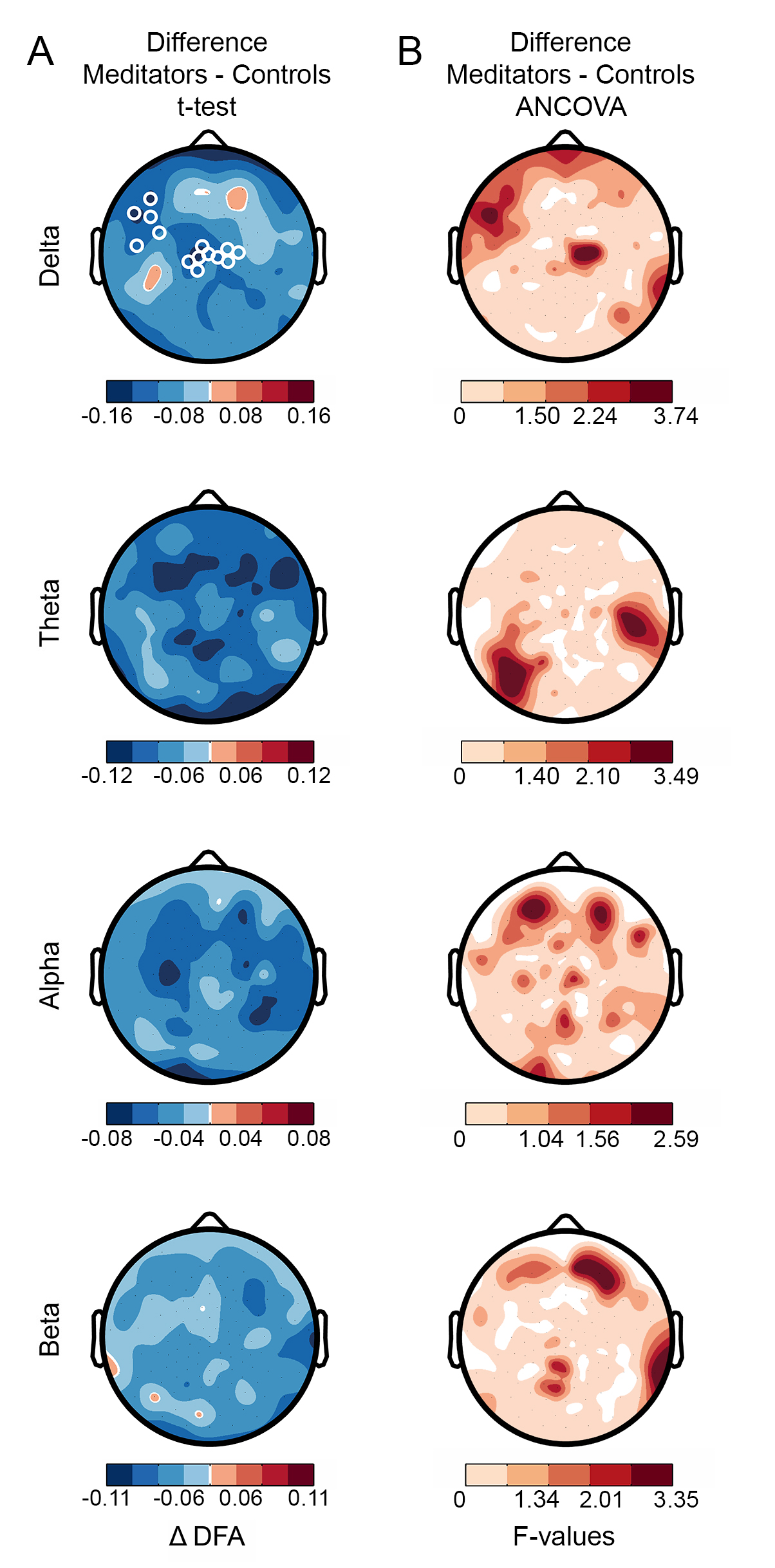

Supplement: Supplementary file 1 — Supporting Information Figure 1 [file HBM-39-1825-s001.tif]

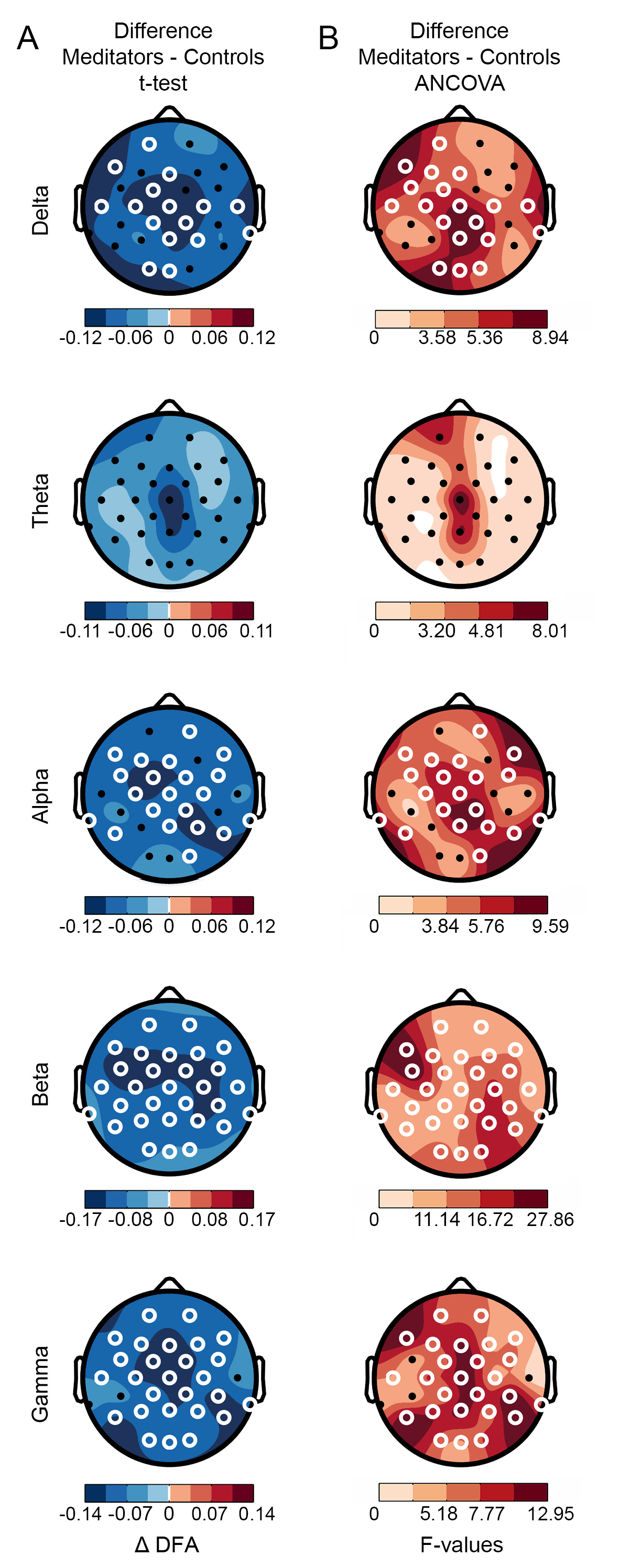

Supplement: Supplementary file 2 — Supporting Information Figure 2 [file HBM-39-1825-s002.tif]
