# Supplementary material for: Controlling the Temporal Structure of Brain Oscillations by Focused Attention Meditation
Source: Hum Brain Mapp. 2018 Jan 13;39(4):1825–38. doi: 10.1002/hbm.23971 (PMC6585826; doi:10.1002/hbm.23971)
Supplement: Supplementary file 3 — Supporting Information Figure 1 [file HBM-39-1825-s003.docx]

**Supporting Information:**

**Supplemental Figure 1: Effect of group on DFA in Study 1**

**A: Grand-average topographies comparing the effect of condition in the meditator and control groups. The difference was calculated as Meditators (MED-ECR) minus Controls (MED-ECR). The rows display DFA for the delta (1–4 Hz), theta (4–8 Hz), alpha (8–13 Hz) and beta band (13–30 Hz), respectively. White circles denote channels with *p* < 0.05 (Independent samples t-test, binomial multiple comparisons corrected). B: Topographies showing the effect of group on DFA in a one-way analysis of covariance (ANCOVA) including the demographic variables Age and Gender. White circles denote channels with *p* < 0.05 (F-test, binomial multiple comparisons corrected).**

**Supplemental Figure 2: Effect of group on DFA in Study 2**

**A: Grand-average topographies comparing the effect of condition in the meditator and control groups. The difference was calculated as Meditators (MED-ECR) minus Controls (MED-ECR). The rows display DFA for the delta (1–4 Hz), theta (4–8 Hz), alpha (8–13 Hz), beta (13–30 Hz) and gamma band (30–45 Hz), respectively. White circles denote channels with *p* < 0.05 (Independent samples t-test, binomial multiple comparisons corrected). B: Topographies showing the effect of group on DFA in a one-way analysis of covariance (ANCOVA) including demographic variables Age and Gender. White circles denote channels with *p* < 0.05 (F-test, binomial multiple comparisons corrected).**
